# Review of Silicon-Based Nanomaterials Fabricated via Laser Microstructuring, Electrochemical Etching, and Hybrid Microstructures: Exploring Functional Integration and Future Applications

**DOI:** 10.3390/nano16211431

**Published:** 2026-11-05

**Authors:** Luis Humberto Robledo-Taboada, Fernando Chiñas-Castillo, Magdaleno Caballero-Caballero, Rafael Alavez-Ramírez, Jesica Ariadna Jiménez-Mendoza, José Raúl Pérez-Higareda, Irving Néstor Sierra-Sánchez, Reynier Suárez Martínez, Laura Elvira Serrano de la Rosa

**Affiliations:** 1 Instituto Tecnológico de Oaxaca, Tecnológico Nacional de México, Calz. Tecnológico No. 125, Oaxaca C.P. 68030, Mexico; luis.rt@itoaxaca.edu.mx (L.H.R.-T.); jose.perez@itoaxaca.edu.mx (J.R.P.-H.); 2 CIIDIR Unidad Oaxaca, Instituto Politécnico Nacional, Hornos No. 1003, Col. Noche Buena, Santa Cruz Xoxocotlán, Oaxaca C.P. 71230, Mexico; mcaballero@ipn.mx (M.C.-C.); ralavez@ipn.mx (R.A.-R.); jjimenezm@ipn.mx (J.A.J.-M.); 3 Laboratorio de Ingeniería Térmica, Instituto Politécnico Nacional, ESIME-Culhuacán, Av. Sta. Ana #1 000, Col. San Francisco Culhuacán, Coyoacán, Mexico City C.P. 04440, Mexico; isierras@ipn.mx; 4 Departamento de Ingeniería Química, CUCEI, Universidad de Guadalajara, Guadalajara C.P. 44430, Mexico; reynier.suarez@academicos.udg.mx; 5 Instituto de Física, Benemérita Universidad Autónoma de Puebla, 14 Sur y Av., San Claudio C.P. 72570, Mexico; lauras@ifuap.buap.mx

**Keywords:** laser microstructuring, LIPSS, spikes, microcraters, porous silicon, hybrid materials

## Abstract

This review examines silicon surfaces produced by femtosecond laser microstructuring and electrochemical etching, as well as their sequential integration. Laser processing creates multiscale topographies, including laser-induced periodic surface structures (LIPSS), conical features, and craters, whereas electrochemical etching generates porous silicon with tunable pore dimensions and high surface area. The central focus is the process coupling between these techniques: how laser-generated topography, defects, and modified surface chemistry may influence subsequent electrochemical dissolution, and how etching, in turn, may alter the stability and wetting of the laser-defined architecture. Available studies demonstrate substantial changes in morphology, roughness, and contact angle after sequential processing, but do not yet establish universal synergistic enhancement or self-cleaning performance. Accordingly, this review distinguishes experimentally demonstrated process-integration effects from mechanistic hypotheses and future design targets. It also evaluates evidence limitations related to durability, mechanical stability, scalability, reference accuracy, and application-specific validation, with particular attention to conceptual sensing platforms based on hierarchical silicon surfaces.

## 1. Introduction

In recent decades, silicon has evolved beyond its conventional role in microelectronics and emerged as a key material for functional surface engineering. Advances in high-precision techniques, such as femtosecond laser ablation and electrochemical etching, have enabled the modification of material morphology at the micro- and nanoscale [1,2]. Porous silicon (PS), produced through controlled electrochemical etching, is a multifunctional material with high surface area, biocompatibility, optoelectronic properties, sensing capabilities, and chemical functionalizability. These attributes make it suitable for applications in gas sensing, controlled drug delivery, and optoelectronic platforms [3,4]. These technologies enable the fabrication of surface structures such as craters, conical pillars, and Laser-Induced Periodic Surface Structures (LIPSS), which exhibit distinctive features including superhydrophobicity, low reflectance, and high molecular adsorption capacity. These characteristics position them as ideal candidates for integration into advanced photonics systems, biomedical devices, and energy conversion applications [5,6].

This review does not address whether laser structuring and electrochemical etching are individually useful, but rather how the first process’s state affects the substrate’s response to the second. Therefore, in this paper, the term “hybrid silicon” refers to a sequentially processed, hierarchical architecture on the same surface. Throughout the review, measured changes are separated from proposed mechanisms and from application targets that have not been validated on the hybrid platform.

Although each technique has matured independently, combining them sequentially offers a potentially useful route to producing hierarchical silicon surfaces with tunable morphology and wettability. Selected studies report hydrophobic or highly water-repellent behavior after combined processing, but the available evidence does not establish universal superhydrophobicity, self-cleaning, or improved biological performance. The principal value of this hybrid approach is therefore a testable structure–property framework, but its device-level advantages, scalability, and durability remain to be demonstrated.

Despite significant advances in laser texturing and electrochemical porosification, the structure–property relationships governing their sequential integration remain poorly understood. Laser processing can introduce multiscale topographies, defects, and localized changes in silicon’s optical and electronic properties, while electrochemical etching modifies the surface through current-mediated dissolution and pore growth. When combined, these processes yield performance that depends not only on each technique’s contribution but also on the sequence, processing window, and interfacial coupling. Consequently, variations in laser fluence, pulse duration, scanning conditions, substrate characteristics, electrolyte composition, current density, and etching time can substantially affect pore geometry, roughness, wettability, optical absorption, and surface stability. Therefore, a systematic analysis of these interdependent parameters is necessary to distinguish genuine synergistic effects from simple additive behavior and to identify the key obstacles to developing reproducible, durable, and scalable silicon hybrid platforms for sensing, photonics, and biomedical applications [7,8,9].

This review examines how laser-induced structuring and electrochemical etching interact in silicon, focusing on how pulse regimes influence defect engineering, dissolution kinetics, and performance. Rather than introducing a new technology, we evaluate the current research landscape, highlight emerging trends, and propose experimentally testable approaches to better control porosity, wettability, and multifunctionality.

## 2. Laser Processing of Solid Materials

Materials processing with ultrashort laser pulses has emerged as an advanced strategy for the controlled modification of the structural, optical, electrical, and chemical properties of solid materials. In particular, femtosecond (fs) lasers deposit energy into extremely confined volumes before significant thermal diffusion occurs. This enables light–matter interactions dominated by nonlinear mechanisms and significantly reduces the heat-affected zone (HAZ), overcoming many limitations of conventional manufacturing and surface modification processes based on continuous-wave lasers or long pulses [10,11].

During the interaction of a femtosecond laser pulse with a solid, initial energy absorption occurs via multiphoton processes and free-carrier generation, particularly in dielectric and semiconductor materials whose bandgap energies exceed the incident photon energy. Rapid electronic excitation can create states far from thermodynamic equilibrium, and energy transfer from the electronic system to the crystal lattice occurs on a picosecond timescale, giving rise to phenomena such as localized melting, ablation, phase modifications, densification, crystal defect formation, and structural reorganization at micro- and nanometric scales [12,13].

One key advantage of femtosecond laser processing is its ability to create three-dimensional micro- and nanostructures with high spatial precision. Because ultrashort pulses deposit energy before substantial heat diffusion occurs, femtosecond processing can reduce the heat-affected zone relative to longer-pulse irradiation; however, a smaller heat-affected zone does not mean the absence of thermal effects. Subsequent electron–lattice energy transfer may cause localized heating, melting, re-solidification, or ablation, depending on fluence and pulse accumulation. Sub-wavelength surface patterns can emerge through coupled electromagnetic energy deposition, feedback, and material reorganization rather than through a universally non-thermal pathway. Among the most widely studied structures are LIPSS, also known as ripples, which form through interference between the incident electromagnetic field and surface waves excited within the material. These nanostructures exhibit periodicities on the order of the laser wavelength or smaller, significantly modifying properties such as reflectivity, optical absorption, wettability, and electromagnetic response [14,15]. Ultrafast lasers can fabricate microstructures, not just surface modifications. The high instantaneous intensity of femtosecond pulses enables nonlinear absorption processes that modify transparent materials internally. This capability has enabled the fabrication of optical waveguides, microchannels, three-dimensional photonic structures, and integrated devices directly within the material’s bulk, without conventional lithographic processes [16]. In dielectric materials such as silica glass, laser interaction induces regions of permanent changes in refractive index associated with densification, structural defects, and chemical bond reorganization [17].

Femtosecond laser processing is particularly important for semiconductor materials because it can locally modify electronic and crystalline properties. Researchers have extensively investigated materials such as silicon (Si), germanium (Ge), gallium arsenide (GaAs), and silicon carbide (SiC) for their technological relevance to electronic and photonic devices and energy conversion systems. Ultrashort-pulse interactions with these materials can induce phenomena such as ultrafast recrystallization, controlled defect generation, band-structure modification, surface nanostructure formation, and enhanced optical absorption through hierarchical surface texturing [17,18].

In particular, femtosecond ablation has proven an efficient strategy for generating micro- and nanostructures on silicon to modify the semiconductor’s optical response. Surfaces textured with LIPSS, cone-like structures, and hierarchical micro- and nanopatterns can significantly enhance spectral absorption through multiple internal reflections and optical confinement. These characteristics have driven applications in photodetectors, sensors, solar cells, and advanced optoelectronic devices [19,20]. Furthermore, precise control over energy deposition via ultrashort pulses enables the design of functional architectures within semiconductors without significantly affecting adjacent regions. This capability is particularly attractive for fabricating next-generation electronic devices, where localized engineering of surface and internal properties is crucial for developing miniaturized, efficient, and multifunctional components. In this context, femtosecond laser processing is a key technological platform for fabricating advanced semiconductor materials, offering micro- and nanoscale resolution, three-dimensional spatial control, and direct modification of physical properties.

Therefore, studying the interaction mechanisms between femtosecond laser pulses and solid materials is an interdisciplinary field spanning ultrafast physics, materials science, nanotechnology, and advanced manufacturing. The ability to generate precisely engineered micro- and nanostructures opens new possibilities for developing functionalized semiconductors, in which laser-induced architectures can control optical, electronic, and thermoelectric phenomena in next-generation devices.

## 3. Laser-Microstructured Silicon: High-Precision Morphology Control

Micro- and nanostructured materials produced by laser ablation have advanced substantially in recent decades and have become essential tools across scientific and technological fields. These structures serve both fundamental and cutting-edge research. Numerous studies have used numerical simulations to elucidate microstructure formation mechanisms and dynamics; the most important parameter is laser fluence, followed by the number of pulses, pulse duration, and wavelength [21,22,23,24]. In this context, the three most commonly used structures resulting from laser-silicon interaction, LIPSS, spikes, and microcraters, are highlighted [25,26]. Surface engineering techniques that produce optically active features, such as light-absorbing or scattering patterns like LIPSS, have found successful applications in advanced biomedical fields, including implants and biosensors. Ongoing developments in this area have enabled new functionalities and broader applications [27,28,29]. Notably, femtosecond laser irradiation of silicon in the mid-infrared spectrum has enabled innovative subsurface modifications and the direct creation of waveguides. These breakthroughs significantly advance next-generation photonic technologies.

Femtosecond laser ablation enables the formation of structures (e.g., LIPSS, conical spikes, microcraters) with exceptional morphological control by precisely tuning pulse fluence, wavelength, and incidence geometry. These textures impart tailored wettability, light-trapping, and biointerface properties. Researchers have applied these structures in advanced optical coatings, superhydrophobic surfaces, and cell scaffolds [30,31]. Although ultrafast laser systems are increasingly available for precision surface processing, large-area reproducibility, manufacturing throughput, and cost-effectiveness remain significant challenges for the industrial implementation of sequential laser–electrochemical silicon structuring.

Recent reviews document the growing use of laser surface texturing to tailor surface morphology, wettability, optical response, corrosion behavior, and tribological performance [32]. Nevertheless, advances in laser technology alone do not demonstrate that femtosecond-laser/electrochemical processing of silicon is affordable, readily scalable, or straightforward to integrate into industrial production. Such claims require application-specific evidence on equipment costs, throughput, process integration, maintenance, safety, and quality control.

Commercial ultrafast fiber-laser systems are currently available in compact configurations designed for precision micromachining, including surface texturing, drilling, and ablation [33]. However, manufacturer-reported specifications and integration capabilities do not independently establish low operating costs, ease of operation by non-specialists, or industrial readiness for sequential laser–electrochemical processing of silicon. These aspects require application-specific evaluation of processing throughput, equipment and maintenance costs, operational safety, reproducibility, and integration with electrochemical treatment.

Although commercial ultrafast-laser sources and systems are more accessible than in the past, the total cost and technical feasibility of large-scale production still depend on the application and configuration. The main unresolved issues include throughput, optical maintenance, wet-process integration, yield, safety, process monitoring, and the cost of downstream functionalization and quality control. Roll-to-roll or wafer-scale compatibility should therefore be treated as a development objective rather than an established capability.

Although laser-source costs have decreased, a new challenge has emerged in the system’s optical components. To preserve the integrity of the ultrashort pulse, the system requires costly, high-quality optics to prevent dispersive effects that can significantly lengthen the pulse and reduce its peak power at the target. Therefore, the modern challenge is not merely the laser itself but optimizing the entire optical train and integrating the laser process with high-throughput wet-etching techniques to develop a continuous, cost-effective, and scalable manufacturing platform.

Femtosecond laser ablation systems use pulses lasting 50–430 fs at wavelengths between 800 and 1030 nm, creating features from 200 nm to 20 μm. Surface topography depends on fluence, as shown in Table 1. Spherical converging lenses with different focal lengths focus the laser beam onto silicon substrates to ablate microstructures [34,35]. At different pulse fluences, microstructures include crater-like features near 1.09 J/cm^2^, conical peaks near 486 mJ/cm^2^, and LIPSS near 220 mJ/cm^2^ [34,35,36,37,38]. This indicates that controlling the formation of a specific microstructure is achieved by adjusting the feed rate or, for large areas, the translation-stage scanning speed relative to the energy density deposited in the material [21,22,23,24,34,35].

In a typical experimental setup for femtosecond laser ablation of silicon, mirrors direct laser pulses to the silicon wafer. An optical attenuator adjusts the pulse energy. An image relay system projects the laser beam waist onto the wafer surface and onto a CCD camera (see Figure 1). The setup also includes an X-Y-Z translation stage to adjust the sample position relative to the laser beam and a pair of goniometers to adjust the angle of incidence. In typical laser ablation experiments on silicon in ambient air at atmospheric pressure, femtosecond-pulsed lasers are employed, with pulse durations ranging from 50 to 430 fs. The laser beam exhibits a Gaussian intensity profile and operates at wavelengths between 500 and 1030 nm [25,34,35,36,37]. Both parameters depend on the specific laser system and the configuration settings defined during fabrication and calibration.

In Figure 1, laser pulses are directed through a series of mirrors to the silicon wafer (optical path A, shown in red). An optical attenuator adjusts the pulse energy. An image relay system (optical path B, shown in blue) images the laser beam waist onto the wafer surface and onto a CCD camera. The setup also includes an X-Y-Z translation stage to move the sample relative to the laser beam and a pair of goniometers to adjust the angle of incidence.

The resulting morphology from the interaction between the laser and the silicon surface is strongly influenced by the energy density (fluence), laser wavelength, and the number of pulses. LIPSS form at low fluences, and their periodicity depends directly on the laser wavelength. These structures are particularly suitable for sensing applications due to their high surface-to-volume ratio and potential for functionalization. Conical structures form at intermediate fluences, significantly reducing surface reflectivity, enhancing optical absorption, and improving the efficiency of devices such as solar cells. These features may also form under specific environmental conditions or in the presence of surface contaminants during ablation [43,44,45,46,47,48]. At higher fluences or with more laser pulses, crater-like microstructures form and exhibit pronounced superhydrophilicity, characterized by an enhanced ability to retain liquids on the surface [30] (see Figure 2).

Beyond conventional control of laser fluence and pulse accumulation, spatial polarization and vectorial optical-field engineering offer additional opportunities for sub-wavelength structuring. Recent studies demonstrate longitudinal-field-enhanced femtosecond-laser patterning of single-crystal silicon [49] and copper [50]. In the earlier copper study, Wang and Li [50] employed radially polarized annular illumination, a double-axicon optical system, and high-numerical-aperture focusing (NA = 0.95) to process a 100 nm copper film deposited on a silicon substrate. They reported a minimum groove-bottom width of 47 nm (approximately λ/22) at a wavelength of 1035 nm, while optical-field calculations indicated a longitudinal-component proportion approaching 98%. Subsequently, Wang et al. [49] investigated annular modulation of radially polarized Bessel–Gaussian femtosecond laser beams for direct structuring of single-crystal silicon. Their numerical simulations predicted a longitudinal-field focal full width at half maximum (FWHM) below 0.2λ, whereas experimental laser scribing yielded nanogrooves approximately 254 nm wide (approximately λ/4) using a 1033 nm laser with a pulse duration of 263 fs. These studies show the potential of optical-field engineering for sub-wavelength structuring of different materials; however, the simulated focal-field dimensions and experimentally measured groove widths should not be equated. Neither study investigated subsequent electrochemical pore formation or demonstrated laser–electrochemical synergy.

These surface morphologies have diverse applications, ranging from designing color-changing surfaces via optical scattering to developing superhydrophobic surfaces, where minimizing water contact with conical tips yields high contact angles and imparts hydrophobic properties [34,38,51,52]. Deep craters generated by laser ablation have also proven effective as cell-culture substrates, owing to their three-dimensional morphology and enhanced biological interactions. Researchers can obtain these microstructures through two primary approaches: increasing the number of laser pulses or raising the incident energy density on the surface [34,35,37,43,48]. Overall, laser microstructuring of silicon is a powerful, versatile tool for designing functional materials across photonics, biomedicine, energy, and surface science.

## 4. Electrochemical Etching of Solid Materials: Formation of Microstructures and Applications in Semiconductors

Electrochemical etching (ECE) is an advanced technique for modifying solid material surfaces, based on controlled oxidation and electrochemical dissolution at the solid-electrolyte interface. Unlike conventional manufacturing methods, this strategy enables selective material removal and the creation of functionalized surfaces with micro- and nanoscale architectures by tuning parameters such as current density, applied potential, electrolyte composition, crystallographic orientation, and mass-transport conditions. This control over morphological evolution has made electrochemical etching a key tool for fabricating microelectronic devices, sensors, photonic systems, and advanced functional materials [53,54].

During the electrochemical process, charge transfer at the solid surface induces oxidation, followed by controlled material dissolution that leads to structural self-organization. Depending on the experimental conditions, researchers can obtain various micro- and nanostructures, including pores, channels, cavities, rough surfaces, and hierarchical architectures with a high surface-area-to-volume ratio. These modifications alter the material’s physical and chemical properties, such as wettability, surface activity, electron transport, optical response, and electrochemical behavior, thereby expanding its potential for technological applications [54,55].

Among the materials studied via electrochemical etching, silicon (Si) is one of the most important semiconductors due to its fundamental role in the electronics and photonics industries. Electrochemical modification of silicon enables the formation of porous silicon (PS) through localized dissolution in electrolytes, primarily hydrofluoric acid (HF)-based. By adjusting current density and process conditions, researchers can control the formation of microporous, mesoporous, and macroporous structures, thereby modifying the material’s electronic, optical, and chemical properties through carrier confinement and increased surface area [56,57].

These silicon micro- and nanostructures form through mechanisms linked to the spatial distribution of electronic holes at the semiconductor-electrolyte interface, where regions of higher electrochemical activity dissolve more rapidly. This behavior enables the fabrication of surfaces with controlled three-dimensional architectures, including multilayer porous layers, optical microcavities, photonic structures, and black silicon-like surfaces that reduce reflectance and increase optical absorption through light-trapping effects and multiple internal reflections [58,59]. In this context, electrochemical etching of silicon is a strategic platform for advanced semiconductor engineering, enabling precise modification of surface morphology and tuning of fundamental properties for applications in photodetectors, chemical sensors, solar cells, and optoelectronic devices. Integrating micro- and nanostructures via electrochemical processes offers a promising pathway to functionalized semiconductor materials with engineered properties, compatible with current demands for miniaturization, energy efficiency, and advanced manufacturing [60,61].

## 5. Porous Silicon via Electrochemical Etching

Porous-material technology relies on creating layered structures with varying porosities, enabling a wide range of applications, from photonic crystals to advanced quantum technologies [62,63]. Researchers obtain porous silicon (PS) by electrochemically etching crystalline silicon, yielding porous networks with controlled geometry. Key variables include substrate doping type (P-type or N-type), crystallographic orientation, electrolyte composition, current density, and electrochemical etching time. For N-type silicon, the process traditionally requires an ultraviolet (UV) light source to facilitate pore formation [4,64]. Conversely, P-type silicon does not require ultraviolet illumination [65,66]; however, studies have also reported pore formation using lasers [67,68]. Laser-assisted electrochemical etching has also been shown to produce nanopores in both silicon types [69]. Forming and controlling a specific pore type does not depend solely on current density.

HF-based electrolytes containing organic solvents such as DMSO or DMF can produce micrometer-scale pores under specific combinations of substrate doping, resistivity, crystallographic orientation, illumination, electrolyte composition, current density, and etching time [70,71]. Square-like pore cross sections have also been reported under certain processing conditions [71], but they cannot be linked to a universal threshold in total anodization current (for example, 200 mA); comparisons require normalization by the exposed electrode area and consideration of interfacial charge transfer and mass transport. Under selected conditions, ethanol-containing electrolytes can produce submicrometer pores [70], but ethanol alone neither determines pore size nor guarantees photoluminescence. In porous silicon, light emission generally arises from the electronic structure of nanoscale silicon domains, including quantum-confinement effects, while surface passivation, oxidation, and defect states can modify its intensity and spectrum [62,63]. Therefore, pore cross-sectional geometry and macropore diameter should be distinguished from the dimensions and chemistry of the nanoscale emitting regions.

In the experimental design for electrochemical etching of porous silicon, a computer with control software typically regulates the power supply. An interconnected multimeter monitors the voltage and current delivered during the process. This setup is connected to a Teflon-based electrochemical cell [4,7,34,64,65]. The anode consists of crystalline silicon attached to a copper plate, while the cathode is a platinum wire designed to withstand high electrical currents. The electrolyte is based on hydrofluoric acid (see Figure 3).

Depending on the fabrication parameters, Table 2 indicates that micropores, mesopores, or macropores, along with various surface morphologies, can be obtained. Reported morphologies include rounded pores and, under certain substrate–electrolyte–anodization combinations, square-like features [70,71]. These outcomes are not universal across low-, intermediate-, or high-current regimes. The absolute current values summarized in Table 2 are specific to the reported experimental setups; meaningful comparisons require current density (based on the exposed silicon area), substrate properties, electrolyte composition, illumination, and etching conditions.

Under the experimental conditions summarized in Table 2, the morphology of electrochemically etched silicon depends on the combined effects of substrate doping and resistivity, crystallographic orientation, electrolyte composition, current density, illumination, and etching time. For example, Robledo Taboada et al. [72] reported rounded macropores at an applied current of 100 mA and square-like macropores at 200 mA, using p-type Si(100) substrates with a resistivity of 5–10 Ω·cm, an HF:DMSO electrolyte (3:7), an exposed area of 1 cm^2^, and an anodization time of 4 min. These conditions correspond to current densities of 100 and 200 mA/cm^2^, respectively. Other electrolyte systems, including HF:DMF mixtures, exhibit different pore-development behavior depending on substrate resistivity and anodization conditions [71]. Consequently, rounded or square-like macropore formation should be interpreted as a condition-dependent experimental result rather than a universal transition governed solely by anodization current.

It is important to distinguish the sub-wavelength nanogrooves obtained by longitudinal-field-enhanced laser scribing, as demonstrated by Wang et al. [49], from the electrochemically generated pore morphologies summarized in Table 2. In laser scribing, material removal is governed by spatially controlled optical energy deposition, whereas electrochemical pore formation depends on interfacial charge transfer, semiconductor properties, electrolyte composition, and anodization conditions. Consequently, the approximately 254 nm laser-written groove width reported in Ref. [49] should not be interpreted as an electrochemical pore diameter or as evidence of synergistic laser–electrochemical processing. Nevertheless, advanced optical-field control may provide an alternative laser-prestructuring strategy for future sequential processing, although its influence on subsequent pore nucleation and growth remains experimentally unverified.

Porous silicon (PS) structures are biodegradable and biocompatible, have high surface area, and are optically tunable, making them ideal for applications such as drug delivery, photonic crystals, gas sensing, and protein-functionalized biohybrids [76,77,78,79,80,81].

Although cost-effective and scalable, PS was previously limited by mechanical fragility and susceptibility to ambient degradation, necessitating surface passivation or hybridization strategies; this is no longer the case. For example, electrochemical alkylation has demonstrably improved the chemical stability of porous silicon by introducing strong Si-C bonds [82]. Studies have also shown that a two-step procedure, functional-group attachment followed by methyl capping, can yield an even more stable material that withstands treatment with chemical oxidants and saline solutions [82]. For biomedical applications, strategies involving zwitterionic poly(sulfobetaine methacrylate) coatings have been developed to enhance resistance to biofouling while maintaining biocompatibility [83].

In medicine, porous silicon stands out for use in advanced wound-treatment systems. Its porous morphology and surface-functionalization capabilities make it a platform for biodetection and controlled drug delivery (pharmaceutical carriers) [84,85,86]. These properties enable real-time monitoring and targeted therapies. In this context, porous silicon also shows strong potential for gas detection because its high molecular adsorption capacity significantly enhances chemical sensors’ sensitivity and selectivity [87,88]. Similarly, in biomedicine, PS nanoparticles produced by electrochemical etching serve as controlled drug-delivery systems, known for their excellent biocompatibility and precise dosing. In addition, researchers have integrated porous silicon into biohybrid materials for optoelectronics and biophotonics [63], where functionalization with photosynthetic proteins has shown remarkable photoactivity, proving useful in low-power biodetection devices and in research on photosynthetic processes [89,90]. In separation technologies, porous silicon (PS) and silicon carbide-based membranes have been successfully applied to reverse osmosis, nanofiltration, and the selective recovery of gases such as hydrogen and carbon dioxide, highlighting their potential for energy and environmental applications. Precise control of porosity, distribution, and morphology through current density and electrochemical etching time enables the fabrication of structures with advanced optical properties, making them ideal candidates for photonic crystal applications [91,92,93]. These applications underscore porous silicon’s multifunctional, versatile, and strategic role in developing high-impact technologies across science, medicine, and energy engineering (see Figure 4).

Silicon surfaces fabricated by sequential laser microstructuring and electrochemical etching can exhibit hierarchical architectures with two distinct structural features at different length scales: laser-defined micro- or nanoscale topography and electrochemically generated pores [94]. In this review, hybrid silicon refers to a sequentially processed surface where these features coexist on the same substrate. This architecture should not be interpreted as a stratified multilayer structure unless cross-sectional characterization demonstrates distinct layers. As Figure 5 schematically illustrates, laser irradiation establishes the initial surface topography and may modify the near-surface structural and chemical state, whereas subsequent electrochemical etching introduces porosity within or around the pre-existing features. The resulting morphology depends on laser parameters, substrate properties, electrolyte composition, current density, and etching time.

In the experimental study reported in Ref. [34], silicon surfaces with laser-induced periodic surface structures (LIPSS), conical features, or craters were electrochemically etched in an HF:DMSO electrolyte (3:7) at an applied current of 30 mA for 4 min (240 s). This sequential treatment produced hierarchical surface morphologies and altered their wettability. However, the observed morphological changes alone do not establish synergistic enhancement or superior device performance. Instead, these results provide a structural basis for investigating potential functional applications, including biosensing and photonic devices, whose performance must be validated experimentally using appropriate control surfaces.

Biosensors: Microtopography and porosity may affect fluid access, analyte transport, and the number of available functionalization sites; improving capture efficiency, sensitivity, and selectivity requires direct device-level validation.

Photonic devices: Hierarchical topography and porosity may modify scattering, light trapping, and the effective optical response; experimental validation using matched samples is needed to establish enhanced photonic performance.

These applications remain prospective because the functional improvements have not been directly demonstrated for the sequentially processed silicon surfaces discussed here. In biosensing, laser-defined topography and electrochemically generated porosity may affect fluid transport, analyte accessibility, and the availability of surface functionalization sites. In photonic systems, hierarchical features may alter scattering, optical confinement, and light trapping. Nevertheless, researchers must demonstrate improvements in sensitivity, selectivity, optical performance, or operational durability on the hybrid substrates and compare them with appropriately matched laser-only and electrochemically etched controls. Similarly, do not equate hydrophobic behavior with superhydrophobicity or self-cleaning without sufficient contact-angle and droplet-mobility measurements. Electrical integration also requires direct characterization of interfacial conductivity, electrode stability, and device-level response [95].

Process coupling mechanism. When laser processing precedes etching, the resulting peaks, valleys, crater rims, roughness gradients, and laser-modified subsurface regions can alter electrolyte access and the local distribution of current or electric field. Defect states and altered surface chemistry may also influence carrier transfer, oxidation, and dissolution kinetics. These effects could produce nonuniform pore nucleation or pore growth and may change the mechanical stability of the laser topography. However, this pathway remains a mechanistic interpretation unless matched laser-only, etched-only, and sequentially processed controls, along with cross-sectional imaging, local electrochemical measurements, spectroscopy, or time-resolved etching data, support it. The reverse sequence, electrochemical etching followed by laser processing, should be treated as a different route because porosity changes optical absorption, thermal transport, and mechanical support.

The laser-generated surface state is treated as the boundary condition for subsequent electrochemical dissolution; the mechanistic links remain hypotheses unless directly validated by matched controls and spatially resolved characterization.

Figure 6 shows cone-shaped hybrid microstructures obtained through laser ablation followed by electrochemical etching; small protrusions are also visible and confer hydrophobicity on the material.

Table 3 should be interpreted as a comparison of reported structures rather than a universal ranking of performance. The available data show that sequential processing can produce a hierarchical surface with roughness and wettability distinct from those of either laser-only or electrochemically etched silicon. However, they do not demonstrate that every hybrid surface is superior to both individual-process controls. Comparisons with black silicon, titanium alloys, or other materials are also limited when substrate, processing atmosphere, surface chemistry, measurement protocol, and durability conditions differ. Claims of synergy are restricted to cases in which matched controls show an improvement beyond an explicit additive or baseline expectation.

For the reported laser-first sequence, laser fluence alters the initial morphology and roughness, while subsequent electrochemical etching increases structural hierarchy and shifts the measured contact angle into the hydrophobic range. The reported hybrid angles of 101–125° overlap with the 120° value listed for electrochemically etched silicon alone; therefore, these values support architecture-dependent wetting rather than a consistent superiority of the hybrid. A static contact angle of 125° is hydrophobic rather than superhydrophobic under the commonly used 150° criterion. Contact angle alone also does not establish self-cleaning, which requires a droplet-transport or contaminant-removal measurement. Friction and wear data for the laser-only and hybrid samples remain unavailable and should be identified as a validation gap.

By specifying the laser wavelength (1030 nm) and pulse duration (270 fs), and by varying the laser ablation fluence to 596, 651, and 753 mJ/cm^2^ (corresponding to LIPSS, cones, and craters), periodic structures are produced with roughness values (Ra) ranging from 50 nm to 1.24 μm and hydrophilic properties (contact angles less than 36°). Interestingly, when these structures undergo electrochemical etching, a secondary process, they evolve into structures with higher roughness coefficients (ranging from 1.5 μm to 2.8 μm) and hydrophobic properties (contact angles greater than 101°) [34,72]. Friction analysis has so far been limited to porous silicon samples [72,96], and the research group has not yet analyzed the tribological behavior of the laser-ablated and hybrid samples.

Regarding optical properties, laser microstructuring and electrochemical etching can each modify absorption, reflectance, and surface morphology [9,10,45,46,64]. A sequentially processed surface may provide complementary control of topography and porosity, but matched samples and identical measurement protocols are required to determine whether its optical response is additive, intermediate, or superior to that of the individual processes.

## 6. Process Coupling in Sequentially Structured Silicon

The sequential integration of femtosecond-laser processing and electrochemical etching should be analyzed as a coupled process rather than as a simple combination of two independent surface treatments. The final architecture depends on the processing order, the state of the laser-modified surface before etching, electrolyte transport through the evolving topography, and the mechanical and chemical stability of the resulting porous structure. The laser-first sequence is therefore a testable process-coupling pathway with several linked stages [34,97].

Reverse-sequence evidence. Evidence for etch → laser processing is limited but not absent. Talbi et al. first fabricated mesoporous silicon by electrochemical etching and then irradiated the porous substrate with picosecond laser pulses, demonstrating that the pre-porosified state supports a distinct laser-induced morphology [98]. This result establishes that the processing order changes the boundary condition, but it does not demonstrate equivalence to, or superiority over, laser → etch processing.

### 6.1. Laser Processing Followed by Electrochemical Etching

1. Laser irradiation creates a nonuniform silicon surface containing peaks, valleys, curvature gradients, multiscale roughness, and laser-modified subsurface regions. Depending on fluence, pulse duration, pulse overlap, scanning conditions, wavelength, and substrate properties, the process may also generate defects, phase changes, redeposition, localized melting, or changes in surface chemistry. These features define the initial boundary condition for the subsequent electrochemical treatment and should not be treated as an ideal flat electrode [34,97].
2. During the subsequent electrochemical etch, the laser-generated topography can alter local electrolyte access, wetting, mass transport, and the distribution of electric field and current density. Peaks, valleys, inclined surfaces, and partially shielded regions may therefore etch at different rates. Local geometry can produce spatially heterogeneous pore nucleation and growth, while liquid retention or restricted access may create differences between exposed and recessed regions.
3. Laser-induced defects and changes in surface chemistry may alter carrier transfer, interfacial oxidation, dissolution kinetics, and the local probability of pore nucleation. The magnitude and direction of these effects are expected to depend on doping type, resistivity, crystallographic orientation, electrolyte composition, current density, illumination, and the chemical state of the surface prior to anodization. Accordingly, laser fluence or current density alone cannot reliably predict the hybrid morphology [34,97].
4. Subsequent electrochemical etching creates macropores and surface protrusions on pre-existing laser-generated LIPSS, conical pillars, or craters, as directly observed by SEM in Ref. [34]. The resulting hierarchical morphologies modify water wettability. However, that study did not quantify pore collapse, delamination, mechanical weakening, or long-term topographic stability; these remain hypotheses that require cross-sectional and durability measurements.
5. The final wettability is governed by the combined effects of surface chemistry, roughness across multiple scales, pore accessibility, liquid penetration, trapped air or liquid, contamination, droplet volume, and measurement protocol. Consequently, higher roughness alone does not guarantee a higher contact angle, superhydrophobicity, self-cleaning, or superior performance. The static contact angle is a descriptive wetting metric; to evaluate self-cleaning or application-specific behavior, hysteresis, sliding angle, droplet transport, contaminant-removal tests, and durability measurements are required [34,72].

The pathway described above is a mechanistic hypothesis. Strong evidence would require matched laser-only, etch-only, and sequentially processed controls, together with cross-sectional imaging, local electrochemical measurements, defect or surface-chemistry spectroscopy, and/or time-resolved etching data. Cross-sectional characterization is especially important because a plan-view image cannot establish whether pores penetrate the laser-modified region, whether a stratified multilayer has formed, or whether the apparent hierarchy is limited to surface roughness. Without these measurements, the reported changes in morphology and wetting should be described as observed process-integration effects, whereas claims about interfacial coupling, universal synergy, self-cleaning, or improved device performance should remain hypotheses awaiting future validation.

Critical interpretation of Table 4. The literature supports sequence dependence, but the evidence base is asymmetric: laser → etch aligns directly with the hybrid definition used in this review, whereas etch → laser and concurrent laser-assisted etching represent different boundary conditions. Accordingly, the present review treats the reverse sequence as comparative evidence of process-order sensitivity, not as proof of reciprocal equivalence or synergy.

### 6.2. Quantitative Framework for Laser–Electrochemical Coupling

A critical distinction in the literature concerns whether the final surface should be viewed as the outcome of two independent modifications or as a potentially coupled, sequence-dependent process. For the sequential laser-first route, the laser step establishes the initial topography, defect population, surface chemistry, and local accessibility of the silicon/electrolyte interface. The electrochemical step then acts on this modified boundary condition. This interpretation aligns with the well-established sensitivity of porous-silicon formation to current density, electrolyte composition, illumination, substrate properties, and etching time, and with broader evidence that porous-silicon sensing performance depends on more than geometric surface-area enhancement alone [101,102].

A first-order description of the local electrochemical removal rate is obtained from Faraday’s law:

***v_etch***_(***x***,***y***,***t***)_ = ***J***_(***x***,***y***,***t***)_
***M_Si***/[***n F ρ_Si***]

where ***v_etch*** is the local silicon-removal rate, ***J*** is the local current density, ***M_Si*** is the molar mass of silicon, ***n*** is the effective electron number for the dissolution pathway, ***F*** is Faraday’s constant, and ***ρ_Si*** is the silicon density. The equation is not intended as a universal kinetic law for porous-silicon formation; rather, it provides a physically transparent variable linking the spatially heterogeneous electrochemical field to morphology evolution. In a laser-modified surface, ***J*** should be treated as ***J***_(***x***,***y***,***t***)_, because local surface orientation, curvature, roughness, defects, oxide/hydride termination, illumination, and electrolyte access can all modify the interfacial conditions. The resulting pore nucleation and growth should therefore be discussed as a spatially heterogeneous response rather than as a single etch-rate value.

This framework leads to a testable process chain:

laser parameters → topography + defects + surface chemistry → electrolyte access and local current distribution → silicon dissolution → pore nucleation/growth → final hierarchical morphology → functional response.

Currently, the literature does not justify assigning a universal positive coupling coefficient to this chain. Instead, this review distinguishes three levels of evidence: E1, direct experimental evidence for sequentially processed silicon; E2, mechanistically supported inference based on established laser or electrochemical behavior; and E3, application hypotheses that require device-level validation. This evidence hierarchy prevents properties measured on laser-only or porous-silicon-only controls from being automatically transferred to the hybrid surface. See Table 5.

Key recommendations include developing roll-to-roll-compatible laser systems for scalable patterning, integrating hybrid surfaces with microfluidics for lab-on-a-chip devices, and systematically studying hybrid surfaces under real-world mechanical, chemical, and environmental conditions.

### 6.3. Integration Versus Synergy: A Criterion for Interpreting Hybrid Performance

The term “synergy” should be reserved for cases in which the interaction between the two processes is demonstrated beyond what would be expected from the individual-process controls. A practical experimental baseline is therefore required before making a synergistic claim. For any measured property P, the minimum control set should include untreated silicon (P0), laser-only silicon (PL), electrochemically etched silicon (PE), and sequentially processed silicon (PLE), prepared and characterized using matched protocols. The quantity ΔPLE = PLE − P0 can then be compared with ΔPL = PL − P0 and ΔPE = PE − P0. Because morphology, wetting, optical response, and electrochemical response are generally nonlinear, do not assume equality or simple addition; instead, use the comparison to determine whether the sequential route produces an interaction that is merely complementary, conditionally non-additive, or demonstrably synergistic.

For wettability, the reported 101–125° contact angles indicate hydrophobic behavior on selected hybrid surfaces but do not demonstrate superiority over electrochemically etched controls near 120°. They therefore support architecture-dependent wetting rather than a universal synergistic enhancement. This interpretation also aligns with the broader porous-silicon literature, where pore morphology, surface chemistry, analyte penetration, and electric-field distribution can influence device response beyond nominal surface area [96,103].

### 6.4. Evidence-Based Experimental Validation of the Coupling Mechanism

The strongest unresolved questions concern the spatially resolved current distribution, the role of laser-induced defects in pore nucleation, and the mechanical stability of laser-defined features during porosification. A rigorous validation protocol should therefore include: (i) top-view and cross-sectional SEM to resolve pore penetration and feature integrity; (ii) AFM or profilometry to quantify multiscale roughness; (iii) Raman spectroscopy and XPS to track laser-induced structural and chemical changes; (iv) current–time or potential–time measurements during etching; and, where available, spatially resolved electrochemical methods or equivalent local measurements; and (v) matched post-etch characterization of porosity, pore dimensions, roughness, wettability, and mechanical integrity. These measurements would enable testing of the proposed process-coupling pathway rather than inferring it solely from final morphology.

A particularly informative experimental matrix is as follows: polished Si → laser-only Si → etch-only Si → laser-first/etch-second Si. The four surfaces should be processed from the same wafer batch whenever possible, using identical cleaning, electrolyte preparation, illumination, droplet protocol, and environmental conditions. Reporting J(t), etch depth, pore size distribution, porosity, roughness, and contact-angle hysteresis would provide a quantitative basis for distinguishing process integration from genuine performance enhancement. This matched-control methodology matters because porous-silicon formation is highly sensitive to multiple processing parameters, and sensor performance cannot be inferred from surface area alone [8,103].

## 7. Applications

For example, research has already shown that combining porous silicon and zinc oxide nanostructures can yield a multicolor photoluminescent spectrum [102]. Other studies have shown that, in multilayer porous silicon structures, the composition and properties of the topmost layer primarily govern photoluminescence [104]. Interdisciplinary efforts across materials science, biomedical engineering, and nanophotonics will be crucial to success.

Laser-textured silicon: Femtosecond lasers can produce extreme nanostructures (ripples, cones) with high initial performance, but these features are susceptible to wear, contamination, and environmental degradation [37]. For hybrid laser–electrochemically processed silicon, long-term wettability retention and biofunctional stability under service-relevant exposure remain insufficiently established; therefore, systematic aging, contamination, and abrasion tests are needed. Improving mechanical robustness (e.g., through harder coatings or hierarchical supports) is an ongoing research need.

Porous silicon: The highly porous network offers biodegradability and biocompatibility but poor chemical stability. Freshly etched porous Si readily oxidizes or corrodes in air or water [4,64,105]. Its mechanical fragility (it crumbles under stress) and sensitivity to ambient moisture limit its lifetime unless passivation or encapsulation is applied [4,64].

Hybrid surfaces: Laser-textured silicon, porous silicon, black silicon, and titanium alloys represent distinct material and process platforms. Their reported properties should not be taken as evidence that hybrid silicon is superior to any of these alternatives unless substrates, metrics, measurement protocols, and durability conditions are matched. A sequentially processed silicon surface may combine laser-defined topography with porous regions, but experimental data are still needed to establish the resulting optical, electrical, sensing, mechanical, and environmental performance. The black-silicon comparison should also distinguish laser processing performed in air from fabrication routes that use fluorinated gases such as SF_6_; the absence of SF_6_ in one air-processing route does not establish a general environmental advantage over all black-silicon fabrication methods.

Future work (shown in Table 6) should include:

1. Photoluminescence characterization of hybrid layers at different laser fluences to assess optical emission stability [102]. The photoluminescent properties of hybrid structures raise promising research questions, such as how the geometric and plasmonic effects of laser-induced periodic surface structures (LIPSS) on a porous silicon layer surface influence the quantum efficiency and spectral properties of its photoluminescence. Such a study could lead to new, spectrally tunable, or more stable light-emitting devices that leverage the potentially complementary optical properties of both the periodic structures and the porous network.
2. Tribological testing under lubricated and dry conditions evaluates the load-bearing capacity and wear resistance of hierarchical surfaces [106]. The tribological effects of laser texturing are well documented [106,107]. Studies on laser-textured surfaces of materials such as silicon nitride have shown that specific patterns can significantly reduce the coefficient of friction and wear [106]. These patterns function by storing lubricants and trapping wear debris, thereby improving the surface’s long-term performance [106]. For example, a research topic could be how combining laser-induced features with porous networks could create a novel tribological platform. Laser-induced microstructures could provide a robust framework for load bearing and wear reduction, while the underlying porous network acts as a lubricant reservoir. This would enable a new class of self-lubricating, low-wear materials.
3. Electrical transport measurements across porous laser interfaces for biosensor integration.
4. Accelerated environmental aging tests (UV, humidity, abrasion) to evaluate hybrid durability relative to TiO_2_ and black silicon analogs.

**Table 6:** Summary of Properties and Proposed Future Research Directions.

Property	State of Existing Research	Proposed Future Research Direction
Tribological Behavior	Studies on laser-patterned surfaces show reduced friction via lubricant trapping and debris removal [106].	Investigate how the hierarchical hybrid structure can serve as a superior tribological surface by combining the load-bearing capacity of laser-ablated microstructures with the lubricant retention of porous networks. Analyses have focused solely on porous silicon and laser-ablated structures; however, no published studies have reported the tribological performance of hybrid structures.
Biocompatibility	PSi is biocompatible, and researchers have developed zwitterionic coatings for anti-fouling [83].	Develop protocols to integrate laser-induced features that enable directional cell adhesion and patterning on porous silicon, creating advanced tissue scaffolds and biosensor platforms. The research team is currently investigating the biocompatibility of porous, laser-ablated, and hybrid surfaces with respect to cells, as well as their antimicrobial behavior; this work raises the possibility of replicating these structures in titanium alloys.
Electrical Conductivity	Not discussed in the paper: a critical property for integrated devices.	The following characterizations remain pending: I–V and resistance measurements; TLM or CTLM (Transmission Line Method/Circular TLM); Electrochemical Impedance Spectroscopy (EIS); c-AFM; KPFM or SSRM (for local conductivity mapping); and Hall effect or Van der Pauw measurements.
Biosensor	Optimization and design of porous silicon-based sensors [108].	This research group is optimizing hybrid-technology sensors; meanwhile, researchers worldwide have proposed various technologies to improve the response of porous silicon as a gas sensor.

## 8. Conceptual Design and Validation Requirements for a Hybrid-Silicon Arsenic Sensor

Scope clarification: This section presents a conceptual design and a proposed validation framework. This study has not fabricated, calibrated, or tested a hybrid arsenic sensor; therefore, detection limit, response time, selectivity, robustness, self-cleaning behavior, and cost are research targets rather than reported results.

Porous-silicon sensors offer a high-surface-area platform for chemical and biological functionalization, but oxidation, humidity sensitivity, and mechanical fragility can limit long-term operation. This section presents a conceptual inorganic arsenic sensor based on a hierarchically structured silicon surface processed in sequence. The proposed combination of laser-defined fluid-routing features and porous regions is a testable design hypothesis; it is not an experimentally validated hybrid arsenic sensor and should not be described as demonstrating self-cleaning or improved environmental resilience.

The proposed device would functionalize the porous region with an arsenic-selective receptor and use laser-defined topography to influence fluid delivery and access to the active area. Porosity may increase the accessible functionalization area, while patterned topography may alter capillary transport; however, these benefits must be verified on the actual hybrid substrate. Integrated electrodes could support impedance or optical readout, but the receptor chemistry, electrode layout, calibration, selectivity, matrix tolerance, and signal transduction remain to be experimentally established.

Various studies have developed sensors based on porous silicon [108,109]. These platforms can be sensitive to oxidation, humidity, fouling, and mechanical damage, but hydrophobicity alone cannot indicate whether self-cleaning is present. This section therefore presents a conceptual arsenic-sensor design based on a hybrid surface. Any proposed environmental-resilience benefit must be verified through contact-angle hysteresis, droplet-sliding or contaminant-removal tests, fouling experiments, and device-level measurements under realistic water matrices.

- Detection limit: A target of ≤1 μg/L (approximately 1 ppb) may be used as a future performance goal because comparable arsenic sensors have reported sub-ppb values under defined conditions. This benchmark comes from non-identical devices, not from the proposed hybrid silicon platform.
- Response time: A response time under 60 s may be set as a design target for field use. Reported response times from other sensor architectures cannot be directly applied to the proposed hybrid device without accounting for sample preparation, receptor kinetics, transport, and readout.
- Cost per test: A low-cost target may be proposed, but the cited graphite-electrode biosensor is not a comparable cost benchmark and does not establish the cost of a hybrid silicon arsenic sensor. The estimate must include wafer processing, laser time, electrochemical etching, functionalization, electrodes, packaging, quality control, reader hardware, and production scale.
- Design hypothesis: Thiol-based or other arsenic-selective receptors could be evaluated in the porous region. The high surface area and capillary transport are expected to facilitate analyte capture, but calibration experiments with As(III)/As(V) speciation, competing ions, pH variation, and environmental water matrices must demonstrate sensitivity and reduced sample volume.

These values should be treated as future validation targets rather than as predictions of the proposed platform’s performance. The porous region may increase surface area, and the laser-defined topography may influence fluid transport, but neither effect establishes a detection limit, response time, robustness, or cost without device-level experiments.

Existing arsenic-sensor studies provide comparative benchmarks for detection limits and response times, but their materials, receptors, electrodes, and operating protocols differ from those of the proposed hybrid silicon design. This review therefore uses those studies to define a validation target and an experimental rationale, rather than to claim that the hybrid sensor has already achieved the reported performance.

- Robustness: The device should work in the field without complex optics. It must resist common interferents (ions, pH changes) and be reusable or low-cost and disposable. Anti-fouling coatings and simple recalibration methods will help meet practical reliability needs.

Finally, the literature on silicon structuring and arsenic biosensors [110,111] offers comparative benchmarks for future validation. A credible cost-per-test analysis would need to account for wafer processing, laser time, electrochemical etching, receptor functionalization, electrodes, packaging, quality control, reader hardware, labor, yield, and production scale.

### Arsenic Sensing as a Validation Case Study Rather than a Demonstrated Device

The arsenic-sensing concept shown in Figure 7 is best treated as a test case for the proposed structure–property framework, not as an established performance claim. Porous silicon is attractive for sensing because pore size, morphology, surface chemistry, and electrical and optical properties can be tuned; however, the sensing response depends on analyte transport, surface functionalization, transduction architecture, and long-term stability rather than on surface area alone [110,111,112]. For the proposed hybrid platform, each causal step should be experimentally validated: laser topography → fluid transport; porosity → accessible functionalization area; receptor chemistry → arsenic selectivity; and electrode/readout → analytical signal. Detection limits, response times, interference tolerance, calibration range, regeneration, and cost per test should be reported only after measurements on the actual hybrid device.

Accordingly, reported sub-ppb arsenic detection limits for other electrode materials should be used only as external analytical benchmarks. They should not be presented as the expected performance of the proposed hybrid silicon architecture. The relevant validation experiments should include As(III)/As(V) speciation, competing ions, pH variation, realistic water matrices, replicate devices, calibration statistics, and aging tests. This approach transforms the arsenic sensor from an unsupported application claim into a clearly defined experimental target.

In published arsenic-sensor architectures, low-ppb detection limits have been reported under optimized conditions [110,111]. For example, the rGO/AuNP/MnO_2_ screen-printed sensor reported a 2.4 μg/L limit of detection for As(III) [112]. Reviews of nanomaterial-based arsenic and heavy-metal sensors indicate that detection limits vary substantially with material, recognition chemistry, transduction mode, and sample matrix [110,112]. Porous silicon platforms offer a large surface area but may undergo oxidation-induced degradation over time [7].

## 9. From the Laboratory to Quality Control

Figure 8 is essential for understanding the fabrication process of the hybrid silicon-based biosensor. The image depicts a series of distinct, interconnected boxes representing each stage of the production process, arranged in a precise left-to-right flow. Arrows connect the boxes, indicating progression from one step to the next and forming a proposed roadmap toward wafer-scale manufacturing of hybrid silicon biosensors; manufacturing readiness has not yet been demonstrated. Each box is labeled with the name of the process step:

1. Laser Texturing: This initial step involves modifying the silicon wafer surface. Below this box, a callout bubble or annotation details the “Risk: Stress-induced defects” and its “Mitigation: Optimized process”.
2. Clean/Passivate: This step focuses on preparing the wafer surface. The associated “Risk: Contamination” and “Mitigation: Cleanroom protocols”.
3. Mask/Electrochemical Etching: This stage involves patterning and electrochemical etching. The “Risk: HF handling” (hydrofluoric acid) and “Mitigation: Fume hood/PPE” are highlighted.
4. Functionalize: In this step, the biosensor is made sensitive to specific biological targets. The “Risk: Bioburden” and “Mitigation: Sterile environment”.
5. Integrate Electrodes/Microfluidics: This involves incorporating the electrical connections and fluidic channels into the device. The “Risk: Delamination” and “Mitigation: Optimized bonding” are addressed.
6. Encapsulate: This step seals and protects the fabricated biosensor. The “Risk: Incomplete sealing” and “Mitigation: Inspection” are listed.

**Figure 8:**
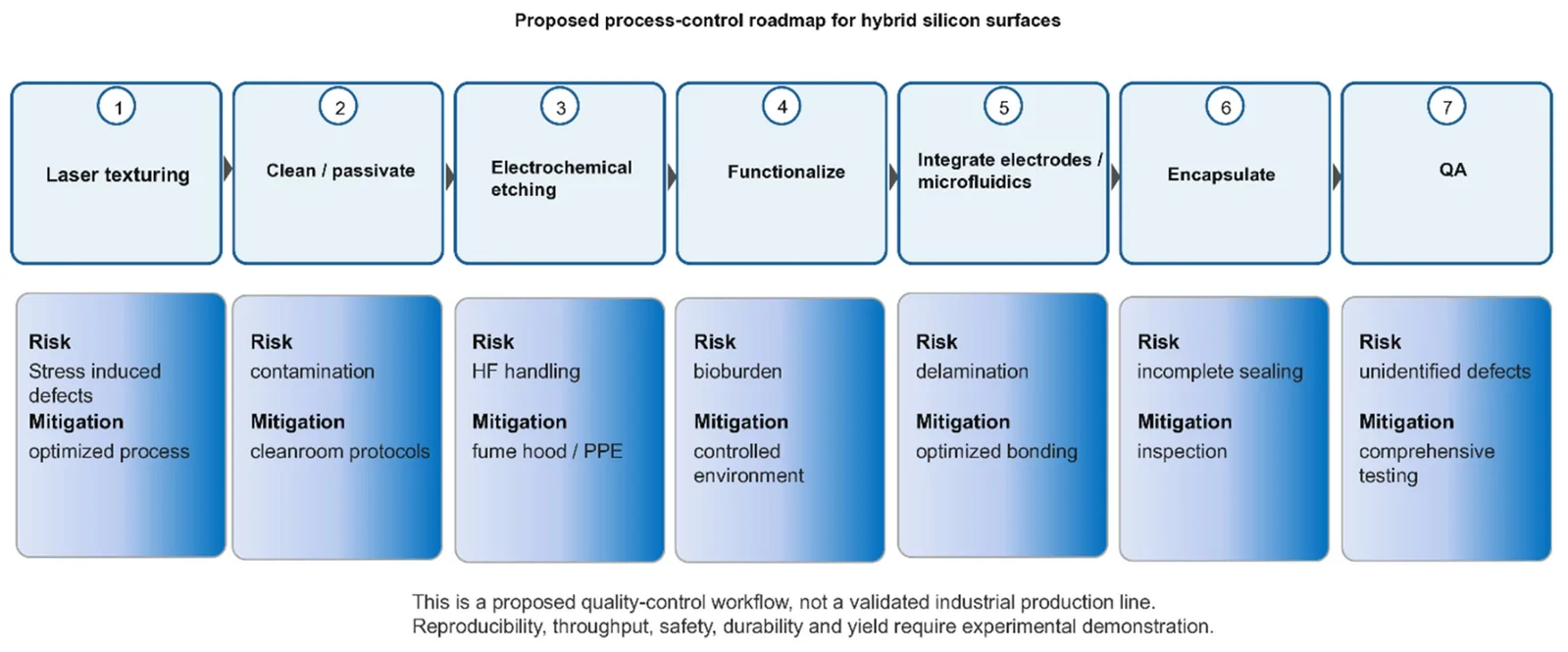
Proposed process-control roadmap toward wafer-scale manufacturing of hybrid silicon surfaces; the workflow is not experimentally validated as an industrial production line.

The roadmap links ultrafast-laser microstructuring and electrochemical porosification as a potential wafer-level workflow, but scalability and reproducibility remain to be demonstrated. Mechanical stress, delamination, HF safety, contamination, and batch variation require standardized process controls and durability metrics. Figure 8 should not imply that the proposed hybrid biosensor has already achieved manufacturing readiness.

Figure 8 presents a proposed quality-control roadmap, not a validated industrial production line. The process sequence should explicitly distinguish laser texturing, cleaning/passivation, electrochemical etching, pore and topography characterization, functionalization, electrode/microfluidic integration, encapsulation, calibration, and batch acceptance. Each stage should be linked to measurable controls, including laser fluence and overlap, current density, pore geometry, roughness, wettability, adhesion, electrical continuity, and sensor response. The roadmap outlines a possible wafer-scale pathway that links ultrafast-laser microstructuring and electrochemical porosification. However, the roadmap has not yet demonstrated manufacturability, reproducibility, durability, safety, or field readiness. Mechanical stress, delamination, HF handling, contamination, batch variation, and sensor calibration require standardized process controls and independent validation before the platform can be considered suitable for industrial or field deployment.

The evidence reviewed here supports complementary control of silicon topography via laser processing and of porosity via electrochemical etching. Sequential processing demonstrably alters morphology, roughness, and wetting in selected studies, but the available data do not establish universal synergistic enhancement, self-cleaning, or superior device performance. The central unresolved issue is the quantitative coupling between laser-induced defects and topography and the kinetics and spatial distribution of subsequent electrochemical dissolution. Future studies should use matched controls, cross-sectional and chemical characterization, local electrochemical measurements, wettability protocols that include hysteresis or sliding angle, and application-specific durability and sensing tests. The inorganic-arsenic sensor remains a testable conceptual design rather than a demonstrated device.

Researchers have demonstrated laser-first/electrochemical-second fabrication of hierarchical silicon surfaces and changes in water contact angle under laboratory conditions, but reproducibility over large areas, throughput, process safety, and cost remain insufficiently established for industrial deployment [34]. Optimizing laser and electrochemical parameters may improve control of morphology and function, but this alone cannot demonstrate economic viability or manufacturing readiness. Long-term stability of the wetting response and surface chemistry under thermal, mechanical, chemical, or outdoor exposure remains insufficiently demonstrated for sequentially processed silicon. Future studies should evaluate photoluminescence, electrical conductivity, biocompatibility, wettability, fouling, wear, and environmental aging using standardized protocols. Potential applications in sensing, microfluidics, photonics, and biomedical devices remain prospective; claims of self-cleaning, sustainability, patentability, or technology transfer require application-specific performance, safety, life-cycle, intellectual-property, and cost analyses.

## 10. Conclusions

Laser processing and electrochemical etching provide complementary control over silicon surface topography and porosity, respectively. Their sequential integration has been shown to modify surface morphology and wettability relative to selected laser-only and electrochemically etched controls. However, the available experimental evidence remains insufficient to establish universal synergistic enhancement or self-cleaning behavior based on the reported contact angles. A key unresolved scientific challenge is to quantitatively determine how laser-induced defects and surface topography influence the kinetics and spatial distribution of subsequent electrochemical dissolution. Addressing this knowledge gap requires systematic investigations using appropriately matched controls, cross-sectional characterization, spatially resolved electrochemical measurements, complementary spectroscopic analyses, durability assessments, and application-specific performance validation. Within this framework, the proposed inorganic arsenic sensor should be regarded as a testable conceptual platform rather than an experimentally demonstrated device.

This review therefore characterizes sequential laser–electrochemical processing as a potentially coupled, sequence-dependent process, with non-additive interactions that remain to be quantified. The first process may modify surface topography, defect populations, and chemistry, thereby influencing the response to the subsequent electrochemical step. The literature currently supports complementary control of morphology and porosity, but it does not establish universal synergy. The most important research opportunity is to quantify local current distribution, defect-assisted dissolution, pore nucleation, and feature stability using matched laser-only, etch-only, and sequential controls. This framework provides a more rigorous basis for designing hybrid silicon nanostructures for sensing, photonics, tribology, and other applications.

The literature supports continued investigation of hybrid silicon as a potential platform for integrating structure and function, but it does not demonstrate universal performance gains over porous silicon, black silicon, titanium alloys, or other platforms. Comparative claims should be limited to matched metrics and conditions, and future work should report application-specific data on optical, electrical, tribological, biological, environmental, and economic performance.

## Figures and Tables

**Figure 1:**
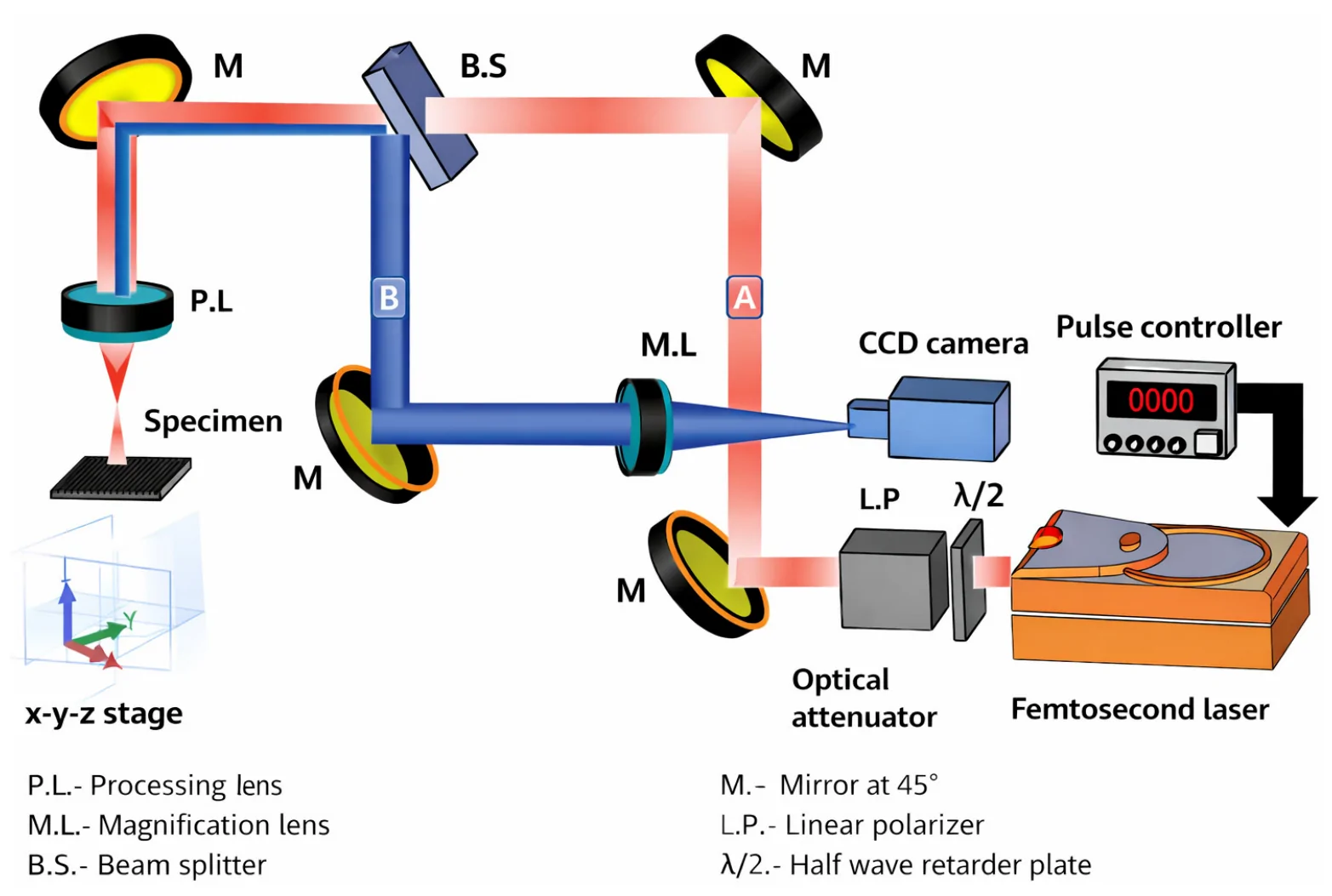
Experimental setup.

**Figure 2:**
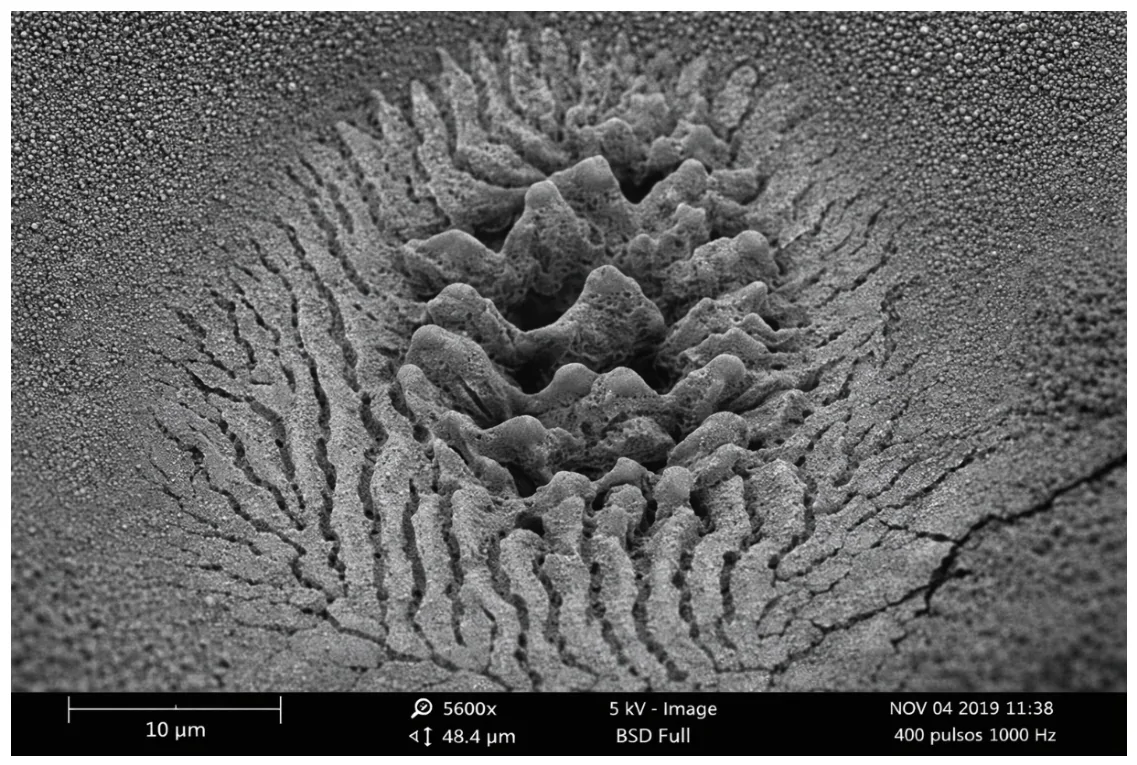
SEM image: Coexistence of LIPSS and conical structures via laser ablation.

**Figure 3:**
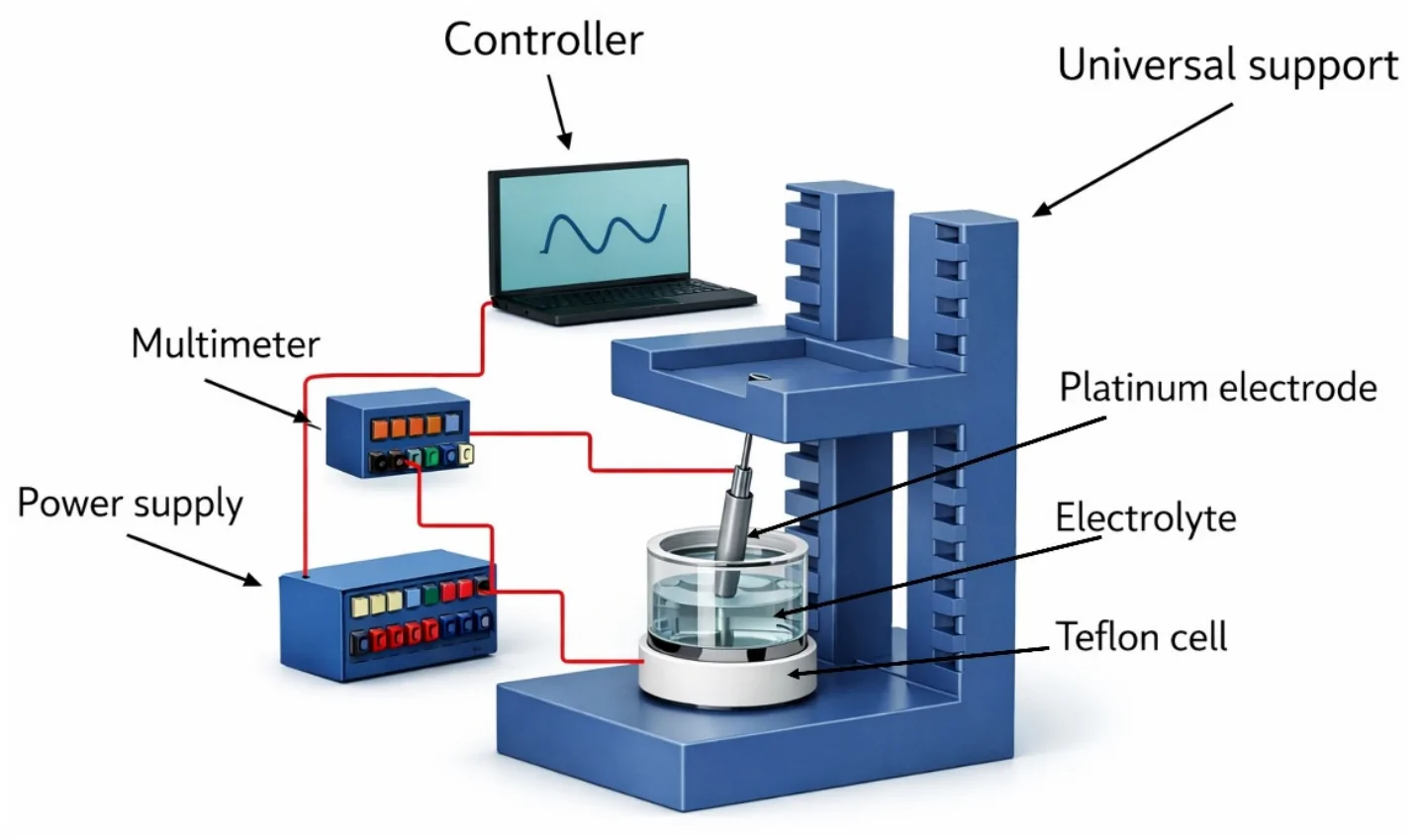
Electrochemical etching setup with a Si anode and a Pt cathode immersed in an HF-based electrolyte, monitored using a computer-controlled current source and a multimeter.

**Figure 4:**
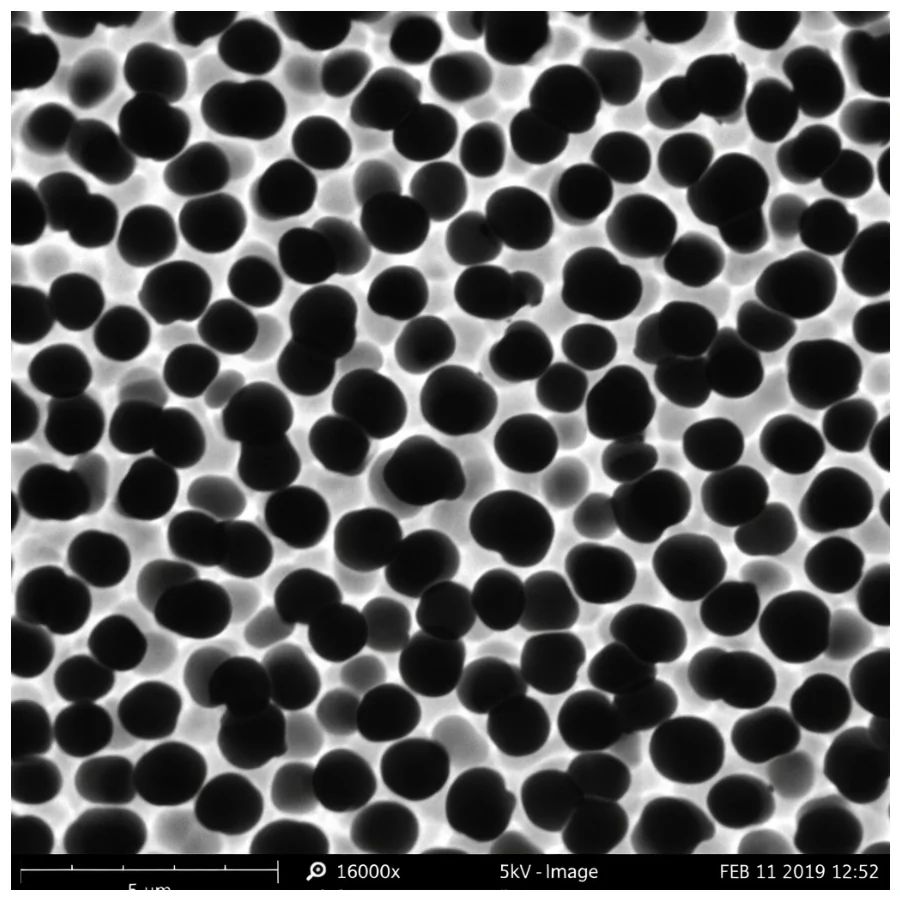
SEM micrograph of macroporous silicon obtained by electrochemical etching using an HF:DMSO electrolyte (3:7) at an applied current of 30 mA.

**Figure 5:**
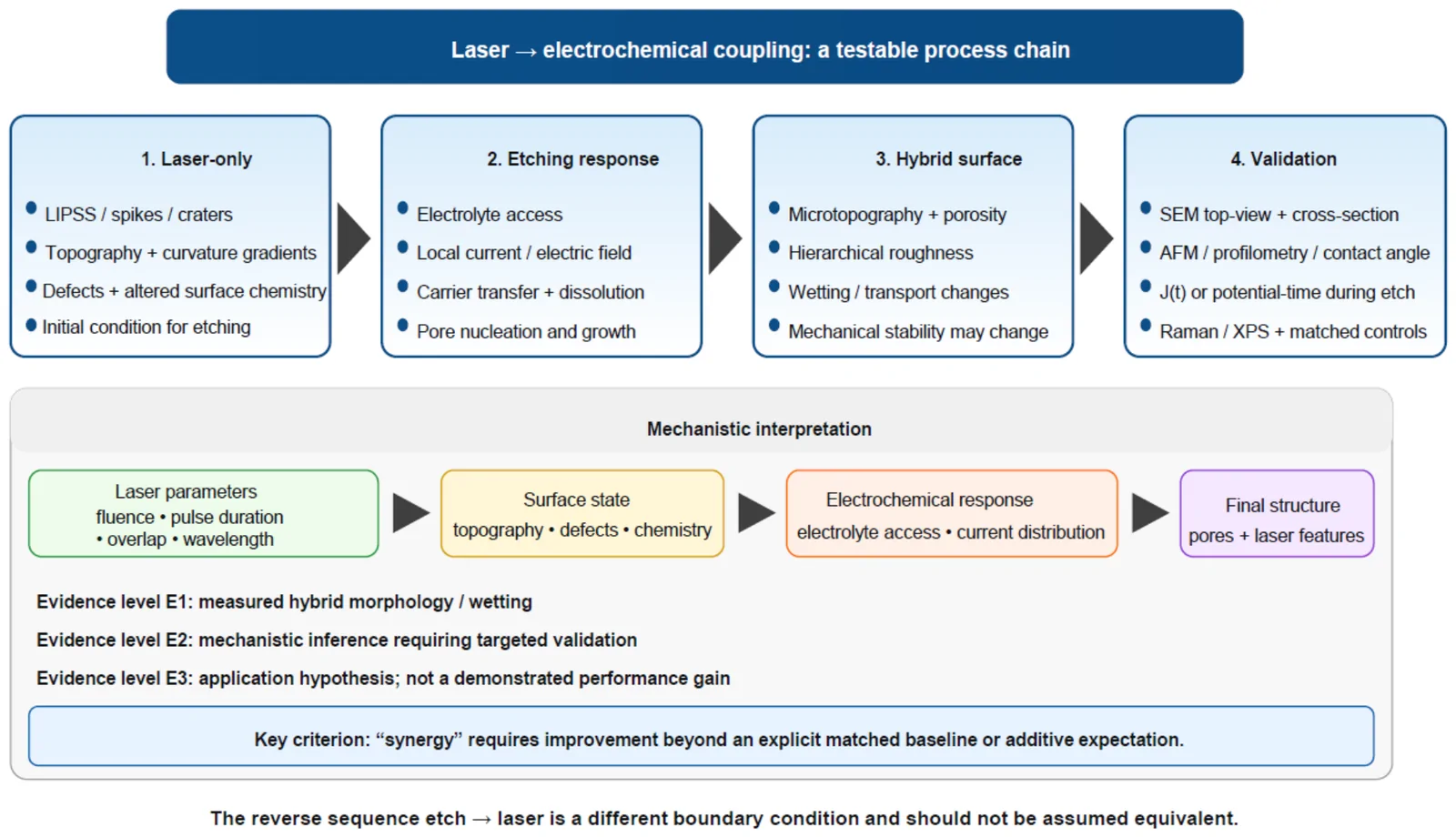
Schematic of the proposed laser–electrochemical coupling mechanism in silicon.

**Figure 6:**
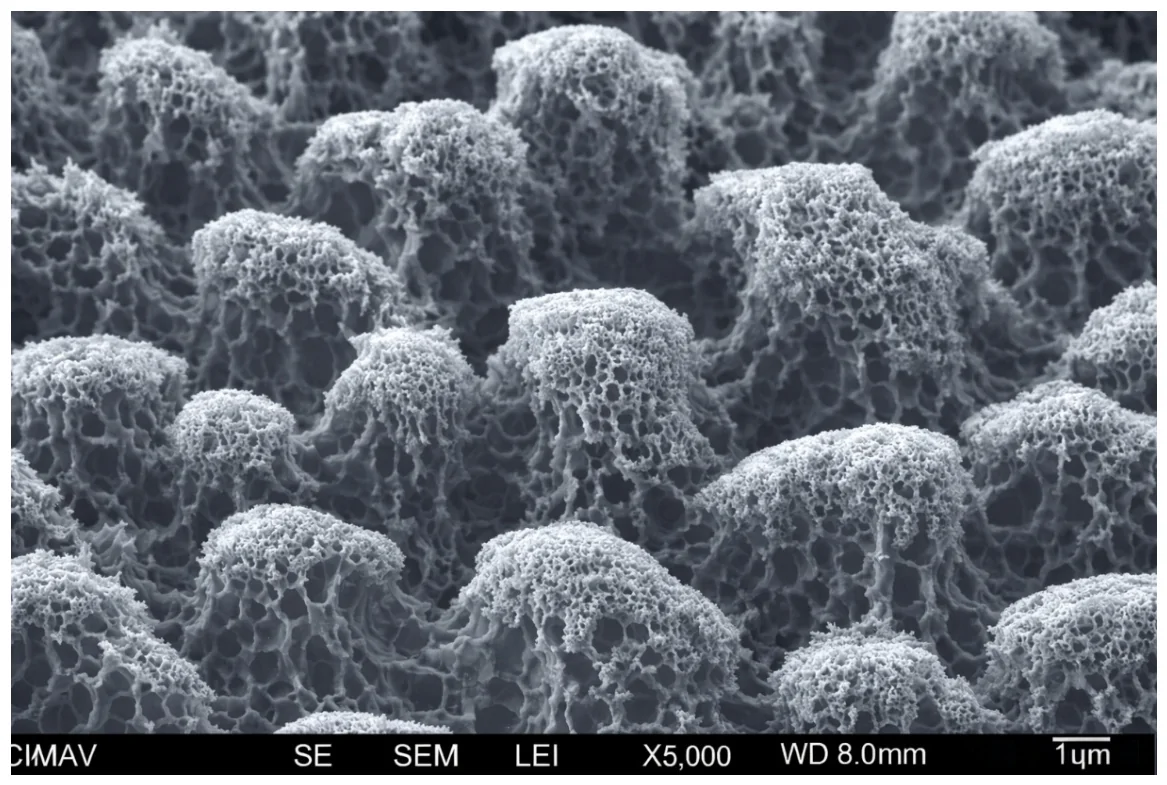
Hybrid architecture showing conical laser-induced micropeaks superimposed on porous macrostructures.

**Figure 7:**
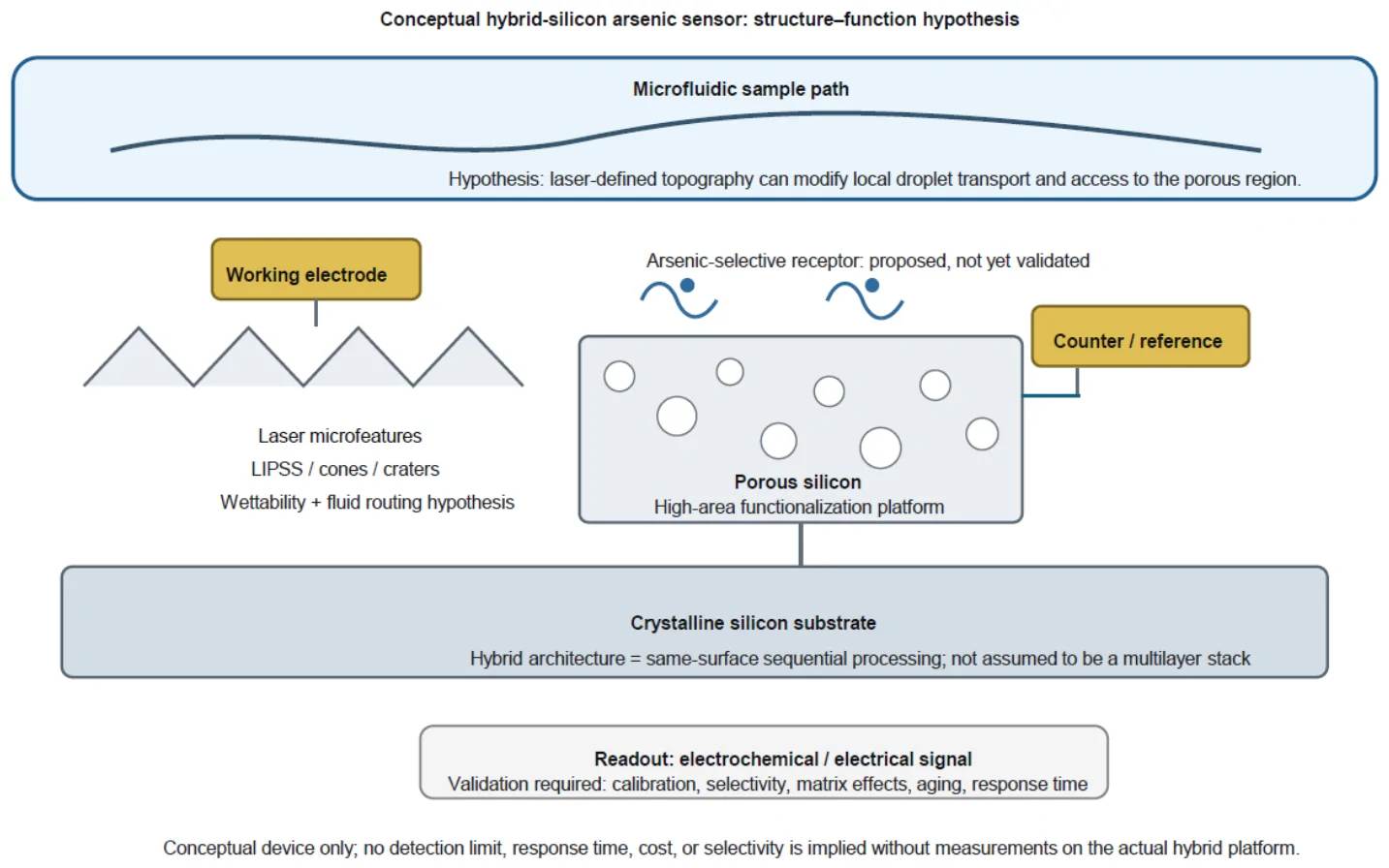
Conceptual hybrid-silicon arsenic sensor integrating laser-defined microtopography, porous silicon, functionalization, and electrical readout. The architecture is a validation hypothesis and does not imply demonstrated analytical performance.

**Table 1:** Laser Processing Parameters for Structured Silicon.

Structure Type	Pulse Duration	Fluence (mJ/cm^2^)	Frequency (kHz)	Pulse Number	Functional Property	Contact Angle	Sweep Speed	Environment
LIPSS [34,35]	270 fs	~550	1	50	High surface area; sensor functionalization	36°	250 µm/s	Air
Conical Spikes [34,35]	270 fs	~640	1	500	Liquid retention; superhydrophilicity	7°	250 µm/s	Air
Deep Craters [34,35]	270 fs	~750	1	900	Liquid retention; superhydrophilicity	4°	250 µm/s	Air
Spikes [39,40]	100–300 fs	200–1500	1–500	-	-	-	1–25 mm/s	Air
LIPSS [41]	10–50 ps	300–2000	50 maximum	-	-	-	2–12 mm/s	Air
LIPSS [42]	5–50 ns	500–5000	10–40	-	-	-	1–1000 mm/s	Air/Liquid

**Table 2:** Study-specific electrochemical conditions and pore morphology.

Si Substrate (Type, Orientation, ρ)	Illumination (Reported)	Electrolyte (Reported)	Etching-Time Effect (Reported)	10–30 mA *	≥100 mA *	>350 mA *	Functional Relevance (Reported/Proposed)
P, (100), 2–30 ohms·cm [71,72]	Natural Light	HF: DMF	Only increases pore depth	Curved pores of 1 to 2 µm	Curved pores of 2 to 3 µm	Square pores with a diagonal of more than 2 µm	Rough surface, light-absorbing
P, (100), 5–10 ohms·cm [72]	Natural Light	HF: DMSO	Only increases pore depth	Curved pores of 1 to 2 µm	Curved pores of 2 to 3 µm	------	Rough surface, light-absorbing
P, (100), 0.05–0.1 ohms·cm [65,73,74]	Natural Light	HF: Ethanol	Only increases pore depth	Pores of 1 to 46 nm	Pores of 1 to 4 nm	Electropolished	Reflective surface, possibility of producing photonic structures
N, (100), (0.001~0.003 ohms·cm) [75]	Ultraviolet	HF: Ethanol	Only increases pore depth	------	------	Electropolished	Surface with photoluminescent, Gas Vapor Sensor

Note. Nominal currents are specific to the cited setups, not universal pore-morphology thresholds. Comparisons require exposure area, substrate properties, electrolyte, illumination, and etching time. Dashes denote entries not tabulated; proposed functions are not validated against device performance.

**Table 3:** (**A**) Processing conditions and structural characteristics of laser-textured, electrochemically etched, and sequentially processed silicon. (**B**) Functional response and strength of evidence for the reported structures.

**(A)**							
**Structure/Sample**	**Process Order**	**Laser Conditions**	**Etch Conditions**	**Morphology**	**Pore Size/Range**	**Height**	**Roughness**
Crystalline silicon [34,35,72]	Untreated baseline	None	None	Polished crystalline surface	Not applicable	0.6995 µm (reported)	10 nm
LIPSS [34,35]	Laser only	Femtosecond laser; 596 mJ/cm^2^	None	Laser-induced periodic surface structures	Not applicable	0.5 µm	50 nm
Spikes/conical structures [34,35]	Laser only	Femtosecond laser; 651 mJ/cm^2^	None	Conical spikes	Not applicable	6.04 µm	1.24 µm
Craters/deep craters [34,35]	Laser only	Femtosecond laser; 753 mJ/cm^2^	None	Deep crater structures	Not applicable	8.2 µm	1.1 µm
Rounded macropores [72]	Etch only	None	100 mA (100 mA/cm^2^), 4 min, HF:DMSO (3:7)	Rounded macropores	1–1.5 µm	Not reported as feature height; porous-layer thickness: 6.31 µm	No directly comparable pre-wear value reported
Squared macropores [72]	Etch only	None	200 mA (200 mA/cm^2^), 4 min, HF:DMSO (3:7)	Square-like macropores	2–3.2 µm	Not reported as feature height; porous-layer thickness: 8.38 µm	No directly comparable pre-wear value reported
LIPSS–macropores hybrid [34]	Laser first, followed by etching	596 mJ/cm^2^; LIPSS formation	30 mA for 4 min; HF:DMSO (3:7) [34]	LIPSS with macropores	Not quantitatively reported for this hybrid structure in Ref. [34]	Not quantitatively reported for this hybrid structure in Ref. [34]	Not quantitatively reported for this hybrid structure in Ref. [34]
Spikes–macropores hybrid [34]	Laser first, followed by etching	651 mJ/cm^2^; conical structures	30 mA for 4 min; HF:DMSO (3:7) [34]	Conical structures with macropores	Not quantitatively reported for this hybrid structure in Ref. [34]	Not quantitatively reported for this hybrid structure in Ref. [34]	Not quantitatively reported for this hybrid structure in Ref. [34]
Craters–macropores hybrid [34]	Laser first, followed by etching	753 mJ/cm^2^; crater structures	30 mA for 4 min; HF:DMSO (3:7) [34]	Cratered hierarchical surface with macropores	Not quantitatively reported for this hybrid structure in Ref. [34]	Not quantitatively reported for this hybrid structure in Ref. [34]	Not quantitatively reported for this hybrid structure in Ref. [34]
**(B)**							
**Structure/Sample**	**Contact Angle**	**Hysteresis/Sliding Angle**	**Optical/Electrical/Sensing Property**	**Control Comparison**	**Evidence Classification**	**Directly Measured in the Hybrid System?**	
Crystalline silicon [34,35,72]	39°	Not reported	Baseline surface response; no application claim	Reference/untreated baseline	Direct baseline measurement	No—untreated/reference control; not a hybrid measurement	
LIPSS [34,35]	36°; hydrophilic	Not reported	Potential for optical structuring and sensor functionalization; device performance not established here	Laser-only sample; matched hybrid comparison not established	Direct morphology and wetting evidence; application relevance inferred	No—laser-only control; not a hybrid measurement	
Spikes/conical structures [34,35]	7°; highly hydrophilic	Not reported	Light absorption and wetting effects; no validated sensing or electrical performance in this comparison	Laser-only sample; not a demonstration of hybrid superiority	Direct morphology and wetting evidence; application relevance inferred	No—laser-only control; not a hybrid measurement	
Craters/deep craters [34,35]	4°; highly hydrophilic	Not reported	Light absorption and surface-liquid interaction; no validated device performance in this comparison	Laser-only sample; not a demonstration of hybrid superiority	Direct morphology and wetting evidence; application relevance inferred	No—laser-only control; not a hybrid measurement	
Curved macropores [34,72]	120°; hydrophobic	Not reported	Rough, light-absorbing surface; no device-level sensing or electrical data reported in the table	Etch-only sample; comparison with laser-only and hybrid samples is not universal	Direct morphology and wetting evidence; functional claims require validation	No—etch-only control; not a hybrid measurement	
Squared macropores [72]	110°; hydrophobic	Not reported	Optical/reflectance implications; no validated sensor performance reported	Etch-only sample; no universal ranking against hybrid samples	Direct morphology and wetting evidence; application relevance inferred	No—etch-only control; not a hybrid measurement	
LIPSS–macropores hybrid [34]	101°; hydrophobic	Not reported	Hierarchical roughness and wetting; no validated optical, electrical, or sensor-device performance demonstrated	Compared with laser-only and electrochemically etched controls for morphology and water contact angle [34]; no measured device-level superiority	Direct hybrid morphology/wetting evidence; device claims conceptual	Partly—hybrid morphology and wetting measured; no device-level optical/electrical/sensing validation	
Spikes–macropores hybrid [34]	115°; hydrophobic	Not reported	Hierarchical roughness and wetting; no validated optical, electrical, or sensor-device performance demonstrated	Compared with laser-only and electrochemically etched controls for morphology and water contact angle [34]; no measured device-level superiority	Direct hybrid morphology/wetting evidence; device claims conceptual	Partly—hybrid morphology and wetting measured; no device-level optical/electrical/sensing validation	
Craters–macropores hybrid [34]	125°; hydrophobic, not superhydrophobic by the 150° criterion	Not reported	Hierarchical roughness and wetting; friction, wear, self-cleaning, and sensor performance remain unvalidated	Compared with laser-only and electrochemically etched controls for morphology and water contact angle [34]; no measured device-level superiority	Direct hybrid morphology/wetting evidence; durability and application claims require validation	Partly—hybrid morphology and wetting measured; no device-level optical/electrical/sensing validation	

Note (A). The rounded and square-like macropore dimensions and anodization conditions reported in Ref. [72] apply to p-type Si(100) substrates with resistivity of 5–10 Ω·cm and an exposed area of 1 cm^2^. The reported current values are specific to these conditions and do not represent universal morphology-transition thresholds. Porous-layer thickness must not be interpreted as feature height, and post-tribological-test roughness parameters must not be directly compared with pre-test surface roughness. (B) Values from different studies are not directly comparable when substrate, surface cleaning, droplet volume, environmental conditions, or measurement protocols differ. Contact angle is reported as a descriptive wetting metric and is not, by itself, evidence of self-cleaning or synergistic performance.

**Table 4:** Critical literature analysis of process coupling between laser microstructuring and electrochemical porosification.

Route	Initial Surface State	Expected Coupling Mechanism	What the Literature Demonstrates	Critical Limitation/Implication
Laser-only	Crystalline Si before electrochemical treatment.	Ultrafast absorption and ablation generate LIPSS, spikes, craters, curvature gradients, defects, and possible changes in chemistry.	Laser processing provides tunable micro/nanotopography and changes optical and wetting behavior.	No electrochemically generated porosity is present in the laser-only surface. Defect population and surface chemistry depend strongly on fluence, pulse overlap, wavelength, and substrate state.
Etch-only	Polished crystalline Si exposed to an HF-based anodic process.	Carrier-mediated dissolution and local current/electric-field distribution control pore nucleation, growth, and morphology.	Electrochemical etching provides tunable pore size, porosity, and surface area; morphology depends on doping, orientation, electrolyte, and current.	The surface lacks the pre-existing laser hierarchy. Mechanical fragility, oxidation and process sensitivity remain important constraints.
Laser → etch	Laser-textured Si enters the electrochemical cell.	Laser topography and defects can modify electrolyte access, local current density, wetting, and dissolution kinetics; etching can subsequently erode or preserve laser features.	Sequential laser-first processing has been experimentally shown to change morphology, roughness, and wettability in hybrid Si surfaces [34].	Direct evidence for universal synergy is absent. Matched laser-only/etch-only/laser → etch controls, cross-sectional imaging and local electrochemical measurements are needed.
Etch → laser	Porous Si is produced first and then irradiated.	The porous substrate changes optical absorption, thermal transport, and mechanical response to the laser; laser irradiation can reorganize or compact the porous surface and form LIPSS.	Talbi et al. first produced mesoporous Si electrochemically and then irradiated the porous substrate with picosecond laser pulses, demonstrating a distinct laser-induced morphology [98].	This sequence is experimentally distinct from laser → etch. Direct reciprocal comparisons remain sparse; reverse-sequence results cannot be used as evidence of laser-first electrochemical coupling.
Laser-assisted/concurrent	Laser irradiation occurs during electrochemical processing rather than as a separate first step.	Direct laser effects, electrochemical dissolution, and liquid-mediated interactions act simultaneously; solution absorption can modify the effective laser–electrochemical coupling.	Long et al. demonstrated coupled laser/electrochemical etching and showed dependence on solution concentration and laser-induced effects [99].	Concurrent laser-assisted etching should not be conflated with sequential laser → etch hybridization because the surface boundary condition evolves while the laser is active.
Mechanistic basis for electrochemical dissolution	Silicon/electrolyte interface with charge carriers and fluoride-containing species.	Surface chemistry, crystallographic orientation and carrier availability constrain the dissolution pathway.	DFT analysis supports a role for direct fluorination and shows that free holes affect the probability of the relevant reactions [100].	This provides mechanistic context for electrochemical etching but does not by itself establish how femtosecond-laser defects alter the subsequent current distribution or pore nucleation.

**Table 5:** Proposed process–structure–property–function framework for interpreting sequential laser–electrochemical silicon structuring. E1 = direct experimental evidence on the hybrid surface; E2 = mechanistically supported inference requiring targeted validation; E3 = application hypothesis.

Laser/Process Variable	Initial State	Electrochemical Consequence to Test	Final Structure/Property	Functional Implication	Evidence Status
Fluence and pulse overlap	LIPSS, cones, craters, defects	Redistribution of electrolyte access and local current	Pore nucleation/growth on a non-planar surface	Control of hierarchical morphology	E2 unless spatially measured
Surface defect density	Defect-rich/chemically modified Si	Possible change in carrier transfer and dissolution kinetics	Localized porosity or preferential dissolution	Potential control of pore placement	E2; requires Raman/XPS + etch kinetics
Topographic curvature	Peaks, valleys, crater rims	Heterogeneous electric-field/current distribution	Nonuniform pore dimensions	Directional fluid access/functionality	E2; requires local electrochemistry
Etching current density	Laser-modified interface	Controls dissolution rate and porosity	Pore size, depth, porosity	Surface-area and transport control	E1 for PSi; E2 for hybrid coupling
Etching time	Evolving hierarchical surface	Progressive pore growth and possible feature weakening	Pore widening, collapse, or preservation	Durability versus accessible area	E1/E2 depending on hybrid dataset

## Data Availability

No new data were created or analyzed in this study. Data sharing is not applicable to this article.
